# Identification of a TIGIT-expressing CD8^+^ T cell subset as a potential prognostic biomarker in colorectal cancer

**DOI:** 10.3389/fimmu.2025.1626367

**Published:** 2025-07-29

**Authors:** Shouli Cao, Meidan Wang, Weiqiang Sun, Zhibin Ma, Kun Yang, Ting Li, Xuedan Zhu, Yang Pei, Mingyue Pan, Liqun Wang, Honglin Ding

**Affiliations:** ^1^ Department of Gastroenterology, Shandong Provincial Third Hospital, Cheeloo College of Medicine, Shandong University, Jinan, Shandong, China; ^2^ Department of Radiation Oncology, Faculty of Medicine, University of Freiburg, Freiburg, Germany; ^3^ Department of Hepatobiliary Surgery, General Surgery, Jinan Third People’s Hospital, Jinan, Shandong, China; ^4^ Department of Gastroenterology The First Affiliated Hospital of Harbin Medical University, Harbin, China; ^5^ Department of Gastrointestinal Surgery, General Surgery, Jinan Third People’s Hospital, Jinan, Shandong, China; ^6^ Department of Oncology, Shandong Provincial Third Hospital, Cheeloo College of Medicine, Shandong University, Jinan, China; ^7^ Faculty of Law, University of Freiburg, Freiburg, Germany; ^8^ Department of Radiation Oncology, Harbin Medical University Cancer Hospital, Harbin, China

**Keywords:** colorectal cancer (CRC), T cell immunoreceptor with Ig and ITIM domains (TIGIT), tumor immune microenvironment (TIME), CXCL13^+^CD8^+^ T cells, PD-1

## Abstract

TIGIT is an inhibitory immune checkpoint receptor on T cells and NK cells that mediates immunosuppressive effects by binding to ligands on malignant or antigen-presenting cells. In colorectal cancer (CRC), immune checkpoint inhibitors like anti-PD-1 show therapeutic promise, but many patients experience resistance or relapse. Therefore, identifying robust immune biomarkers for predicting disease progression and therapeutic response is critical. Analysis of transcriptomic data from CRC patients revealed that high TIGIT expression is associated with poorer overall and disease-free survival. TIGIT expression also correlated with immune infiltration, particularly CD8^+^ T cells. Single-cell RNA sequencing identified a distinct subset of TIGIT^+^PD-1^+^CXCL13^+^ CD8^+^ T cells enriched in CRC patients. *In vitro* co-culture experiments confirmed that this phenotype is induced by tumor cells, suggesting a tumor-driven mechanism of T cell dysfunction. This TIGIT^+^PD-1^+^CXCL13^+^ CD8^+^ T cell population may serve as a potential biomarker for prognosis and immunotherapy response in CRC.

## 1 Introduction

Colorectal cancer (CRC) remains one of the most lethal malignancies worldwide, with an estimated 52,900 deaths projected in 2025 (1). The current standard treatment typically involves surgical resection followed by multimodal therapies, including chemotherapy, immunotherapy, and targeted agents such as epidermal growth factor receptor (EGFR), vascular endothelial growth factor receptor (VEGFR), or human epidermal growth factor receptor 2 (HER2) inhibitors (2–4). Despite significant therapeutic advances, many patients ultimately develop disease recurrence or resistance, particularly in advanced or refractory stages characterized by distant metastases (5). In these cases, treatment options become increasingly limited, and patients may be enrolled in clinical trials investigating novel therapeutic approaches such as bispecific antibodies, emerging immune checkpoint inhibitors (ICIs), chimeric antigen receptor T (CAR-T) cell therapies or target drugs (2, 6–8). Therefore, early detection and the identification of reliable prognostic biomarkers are critical to improving clinical outcomes and guiding personalized treatment strategies (2, 4).

Immune checkpoint blockade (ICB) has revolutionized cancer therapy by restoring antitumor immunity, particularly in tumors with robust infiltration of cytotoxic CD8^+^PD-1^+^ T cells. ICIs targeting the PD-1/PD-L1 axis have shown significant improvements in overall survival (OS) and progression-free survival (PFS) across multiple cancer types (9, 10). In CRC, the efficacy of immunotherapy has been observed predominantly in a subset of patients, particularly those with microsatellite instability-high (MSI-H) or mismatch repair-deficient (dMMR) tumors (11, 12). Moreover, patients diagnosed at earlier stages tend to exhibit better responses to ICB, translating into prolonged disease-free survival (DFS) (13). This underscores the need for early diagnosis and effective biomarkers to stratify patients likely to benefit from immunotherapeutic interventions (14, 15).

TIGIT (T cell immunoreceptor with Ig and ITIM domains) is an inhibitory immune checkpoint receptor expressed on natural killer (NK) cells and T cells (16). It binds to its ligands CD155 (PVR) or NECTIN-2 (CD112), which are frequently overexpressed on malignant or antigen-presenting cells (APCs), TIGIT suppresses cytotoxic immune responses, contributing to immune evasion within the tumor microenvironment (TME) (17, 18). Recently, TIGIT has emerged as a promising immunotherapeutic target across various malignancies due to its association with T cell exhaustion and poor prognosis, prompting the initiation of several clinical trials targeting the TIGIT pathway (16, 19).

In this study, we reveal that the infiltration of CD8^+^TIGIT^+^CXCL13^-^T cells is significantly enriched in the CRC tumor microenvironment and correlates with poor clinical outcomes. These cells exhibit features of exhaustion and are localized primarily within tertiary lymphoid structures (TLSs), suggesting a specialized role in shaping the immune landscape of CRC. Our findings highlight this unique T cell subset as a potential prognostic biomarker and therapeutic target in CRC, providing novel insights into the immune regulatory mechanisms at play and offering new avenues for precision immunotherapy.

## 2 Experiments: methods and materials

### 2.1 Cell culture and *in vitro* T cell activation

MC38 cells, a murine colon adenocarcinoma cell line, were obtained from the American Type Culture Collection (ATCC, Manassas, VA, USA) and cultured in accordance with ATCC-recommended protocols.

For *in vitro* T cell activation, 48-well culture plates were coated with anti-mouse CD3 monoclonal antibody (5 μg/mL; BioLegend, #100201) and incubated overnight at 4°C (Day 1). On Day 2, spleens were harvested from naïve C57BL/6 mice, and single-cell suspensions were prepared via mechanical dissociation, followed by red blood cell (RBC) lysis using RBC lysis buffer. CD8^+^ T cells were subsequently isolated using magnetic CD8a^+^ T cell isolation beads (Miltenyi Biotec, #130-104-075) according to the manufacturer’s instructions. Purified CD8^+^ T cells were resuspended at a density of 5 × 10^5^ cells/mL in complete RPMI-1640 medium supplemented with 10% fetal bovine serum (FBS), 1% L-glutamine, 1% Penicillin–Streptomycin, 1% non-essential amino acids (NEAA), 1% HEPES buffer, 1% 2-Mercaptoethanol, and an additional 1% GlutaMAX. Anti-mouse CD28 monoclonal antibody (2 μg/mL; BioLegend, #102102) was added to provide co-stimulatory signaling.

On Day 3, recombinant mouse IL-2 (10 ng/mL; Thermo Fisher, #212-12-100UG) and IL-7 (10 ng/mL; Thermo Fisher, #217-17-100UG) were added to further support T cell activation and expansion.

On Day 4, MC38 tumor cells were added to the activated T cells at a 1:10 ratio (tumor cell: T cell), and co-culture was maintained for 48 hours under standard culture conditions (37°C, 5% CO_2_). After incubation, cells were harvested for flow cytometric analysis to evaluate the expression of immune checkpoint molecules, including TIGIT and PD-1, on CD8^+^ T cells.

### 2.2 Flow cytometry for TIGIT and PD-1 and the cytokines expression

Following the 48-hour co-culture of the *in vitro* activation T cells and MC38, cells were harvested and washed twice with phosphate-buffered saline (PBS). Surface staining was performed in FACS buffer (PBS supplemented with 2% fetal bovine serum) containing the viability dye Propidium Iodide (PI; Thermo Fisher Scientific, #R37169) and the following fluorochrome-conjugated monoclonal antibodies: anti-mouse CD8-BV421(Biolegend#100737), anti-mouse TIGIT–PE (BioLegend, #142101) and anti-mouse PD-1–APC (BioLegend, #135209). In addition, levels of granzyme B (Biolegend, #396409), and tumor necrosis factor-alpha (TNF-α) (BioLegend, #506315) were measured to evaluate the cytotoxic effects of *in vitro* T cell stimulation, both in the presence and absence of tumor cell co-culture. Surface staining was carried out for 30 minutes on ice in the dark. After washing, Intracellular staining was carried out according to the manufacturer's protocol (Invitrogen, #00-5523-00) cells were resuspended in a FACS buffer and analyzed using a BD LSRFortessa™ flow cytometer (BD Biosciences). Data acquisition was followed by analysis using FlowJo software (version 10.8.1, BD Biosciences). The isotype controls for TIGIT-PE and PD-1-APC are PE Mouse IgG1, κ Isotype Control (BioLegend #981804) and APC Rat IgG2a, κ Isotype Control Antibody (BioLegend #400511), respectively.

### 2.3 Single-cell RNA sequencing analysis of TIGIT, PDCD1, and CXCL13 expression

Publicly available Sc sequencing datasets from colorectal cancer patients and (GSE1467711) were obtained from the Gene Expression Omnibus (GEO) database. Data preprocessing, including quality control, normalization, and scaling, was performed using the Seurat package (version 4.0) in R. Principal component analysis (PCA) and Uniform Manifold Approximation and Projection (UMAP) were applied for dimensionality reduction and clustering. The expression patterns and co-localization of TIGIT, PDCD1, and CXCL13 within CD8^+^ T cell subsets were visualized using feature and box plots in the TISCH 2.0 (Tumor Immune Single-cell Hub, http://tisch.comp-genomics.org) database. Additionally, the GSE178341 from Single cell portal (Tumor Immune Single-cell Hub, https://singlecell.broadinstitute.org/single_cell) database was used to validate co-expression patterns in multiple CRC scRNA-seq datasets.

### 2.4 Immune cell infiltration and deconvolution analysis

To evaluate tumor immune microenvironment composition, bulk RNA-seq data from CRC patient cohorts were analyzed using CIBERSORT-ABS and QUANTISEQ algorithms via the TIMER2.0 platform. CIBERSORT-ABS estimates absolute immune cell fractions, while QUANTISEQ offers robust profiling of immune cell types in solid tumors. Correlation analysis between TIGIT expression and the abundance of tumor-infiltrating immune cells—particularly CD8^+^ T cells—was performed using Pearson correlation coefficients. Results were visualized via dot plots. Receiver operating characteristic (ROC) analysis was performed using the pROC package in R to assess the prognostic value of TIGIT expression.

### 2.5 Co-expression network analysis of TIGIT-associated genes

Gene co-expression analysis was conducted using both the Single Cell Portal and TISCH2.0 database to explore genes significantly correlated with TIGIT in CRC-derived CD8^+^ T cell subsets. Co-expression networks were constructed focusing on genes involved in T cell exhaustion, chemokine signaling, and immune metabolism. Specific emphasis was placed on the identification of functional gene modules co-expressed with TIGIT, including PDCD1 and CXCL13, across healthy donors and CRC patients. Bubble plots representing gene expression differences between conditions were obtained from TISCH 2.0.

### 2.6 Tumor immunity estimation and immune signature correlation

The relationship between TIGIT expression and immune cell infiltration was further validated using the Tumor Immune Estimation Resource (TIMER) and GEPIA2 (http://gepia2.cancer-pku.cn) platforms. The correlation between TIGIT and gene signatures indicative of CD8^+^ T, CD4+ T and nature killer (NK) cell functional states—such as effector, memory, and exhausted phenotypes—was evaluated. Log_2_-transformed TPM (transcripts per million) values were used for statistical correlation analyses.

### 2.7 Cell-cell interaction analysis

Intercellular communication networks within the CRC tumor microenvironment were reconstructed using the TISCH2.0 database and the CellChat R package. Ligand–receptor interaction analyses focused on the inhibitory axes involving TIGIT–PVR(CD155) and TIGIT–NECTIN-2 (CD112). Heatmaps, circle plots, and bubble charts were generated to illustrate cell-type-specific interaction strengths. Special emphasis was placed on the interaction between exhausted CD8^+^ T cells and tumor/stromal cells, and the relative contribution of each population to inhibitory signaling pathways was quantitatively inferred.

### 2.8 Statistical analysis

All *in vitro* experiments were performed in biological triplicates unless otherwise indicated. Data are presented as mean ± standard error of the mean (SEM). Two-group comparisons were conducted using unpaired two-tailed Student’s t-tests. P-values < 0.05 were considered statistically significant (*p < 0.05; **p < 0.01; ***p < 0.001). Statistical analyses were carried out using GraphPad Prism (version 9.0, GraphPad Software, CA, USA).

## 3 Results

The Results section presents a multi-dimensional analysis of TIGIT expression and its immunological implications in colorectal cancer (CRC). Through single-cell and bulk RNA-sequencing datasets, we identify TIGIT as a key marker of exhausted CD8+ T cells. We then explore the expression of its ligands across tumor and stromal cells, investigate clinical correlations with patient prognosis, and validate TIGIT-associated immune interactions. The section concludes with experimental evidence that tumor cells drive TIGIT upregulation, supporting its functional role in T cell exhaustion.

### 3.1 TIGIT is highly expressed on exhausted CD8^+^ T cells and correlates with immune suppression in the CRC tumor microenvironment

**Figure 1:**
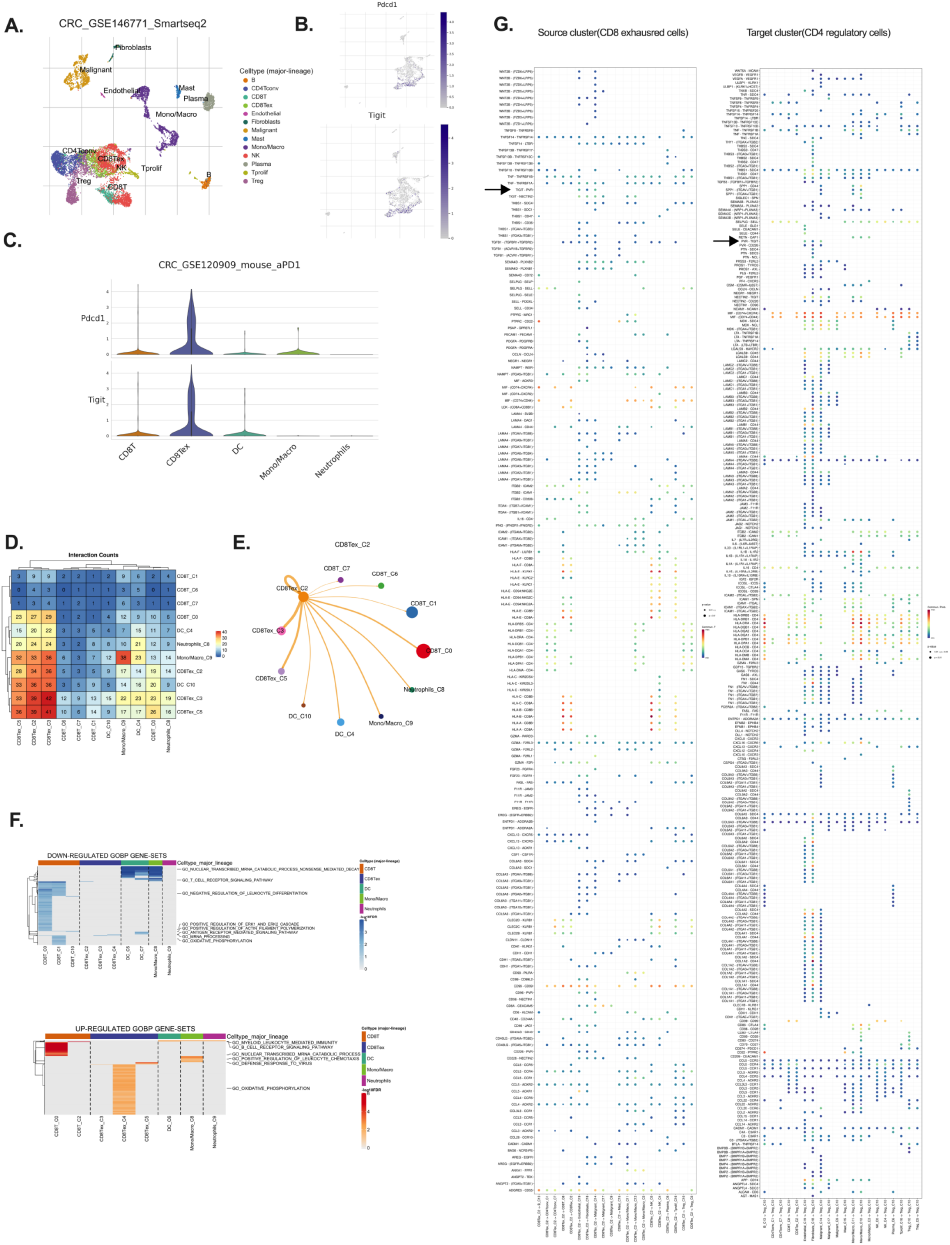
TIGIT is predominantly expressed by exhausted CD8^+^ T cells in CRC. **(A)** Clustering of single-cell RNA-seq data (GSE146771) identifies major immune populations including CD8 T cells, CD8 Tex cells, DCs, NK cells **(B)** Expression distribution of PDCD1 and TIGIT across identified immune clusters; **(C)** Violin plots showing PDCD1 and TIGIT expression levels across immune cell types; **(D)** Differentially expressed genes highlighting upregulation and downregulation in exhausted CD8^+^ T cells; **(E, F)** CCInetworks among immune populations, focusing on inhibitory signaling, particularly on CD8 Tex; **(G)** Source–target mapping revealing exhausted CD8^+^ T cells as major communication initiators; source cluster: CD8 Tex, target cluster: CD4 regulatory T.

TIGIT was significantly upregulated in tumor-infiltrating immune cells in CRC, with predominant expression observed in exhausted CD8^+^ T cells (CD8^+^ Tex) (19). scRNA-seq data from CRC patients (GSE146771) revealed a strong co-localization of TIGIT and PD-1 (PDCD1) within the CD8^+^ Tex subset (**Figures 1A–C**), indicative of a terminally exhausted phenotype (20). Analysis of the overall immune landscape in CRC samples revealed that the predominant immune cell types included CD4^+^ conventional T cells, Tregs, CD8^+^ T cells, CD8^+^ Tex cells, monocytes/macrophages, and natural killer (NK) cells (**Supplementary Figures S1A, B**). Violin plots further demonstrated that TIGIT expression was largely restricted to immune cell populations, with the highest levels detected in regulatory T cells (Tregs) and CD8^+^ Tex cells (**Supplementary Figures S1C, D**) (21, 22).

TIGIT is known to exert its immunosuppressive effects via binding to its ligands, PVR (CD155) and NECTIN-2 (CD112), which are commonly expressed on antigen-presenting cells and tumor cells, thereby attenuating cytotoxic T cell responses (16).Cell–cell interaction (CCI) analysis revealed extensive crosstalk between CD8^+^ Tex cells and other immune populations, including dendritic cells, neutrophils, and Tregs (**Figures 1D, E**). Notably, strong interaction scores were observed between CD8^+^ Tex cells and Tregs via the TIGIT–PVR axis, suggesting that this pathway plays a pivotal role in reinforcing immunosuppressive signaling within the tumor microenvironment. These findings highlight the immunoregulatory centrality of CD8^+^ Tex cells in shaping the TIME through TIGIT-mediated inhibitory interactions (5, 18, 23).

To further explore the functional impact of TIGIT expression, pathway analysis of CD8+ Tex cells demonstrated downregulation of cytotoxic cytokine production, mRNA processing, and antigen receptor signaling, alongside altered oxidative phosphorylation (OXPHOS) (**Figure 1F**) (24, 25). Interestingly, while many functional pathways were suppressed, OXPHOS-related genes were paradoxically upregulated, supporting the notion that exhausted T cells attempt to maintain mitochondrial metabolism despite diminished effector function (**Figure 1F**) (26). Further CCI network visualization demonstrated substantial overlap and connectivity among TIGIT^+^ CD8^+^ Tex cells and other major immune populations in CRC, including Tregs and NK cells, through shared inhibitory circuits (**Figure 1E**). Additionally, analysis of the source–target relationship between CD8^+^ exhausted cells (source cluster) and CD4^+^ regulatory cells (target cluster) reveals a prominent TIGIT–PVR interaction. This source–target mapping highlights exhausted CD8^+^ T cells as major initiators of cellular communication, with the TIGIT–PVR axis playing a crucial role in their interaction (**Figure 1G**).

### 3.2 Stromal and malignant cells cooperatively contribute to TIGIT-mediated immunosuppression

**Figure 2:**
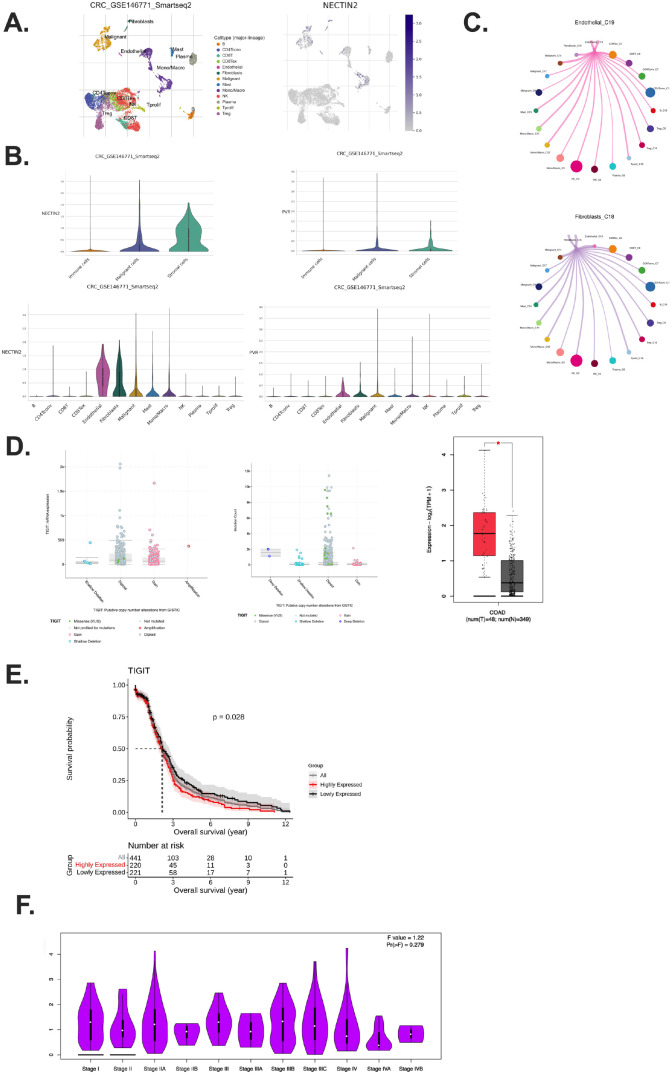
Stromal and malignant compartments sustain TIGIT signaling and associate with poor prognosis. **(A)** Visualization of stromal clusters and NECTIN-2 expression patterns; **(B)** NECTIN-2 and PVR expression across stromal, immune, and tumor compartments in CRC tumor samples; **(C)** Inhibitory interactions with CCI analysis between ECs and malignant cells; **(D)** Relationship between TIGIT expression and mutation burden in CRC patients across CRC patients; **(E)** Kaplan–Meier analysis showing Stratified by TIGIT expression in CRC patients; **(F)** TIGIT expression across different CRC pathological stages from stage I to stage IV. Exhausted T cells (Tex); Dendritic cells (DCs); Nature killer (NK); Cell–cell interaction (CCI).

Given TIGIT’s role in T cell inhibition, we next investigated the cellular sources of its ligands. Using ScRNA seq data (GSE146771) of CRC pre-clinical model, we found that NECTIN-2 and PVR, the two primary ligands of TIGIT, are predominantly expressed by stromal cells, especially endothelial and fibroblast populations (16, 20, 23), particularly endothelial and fibroblast cells (**Figures 2A, B**). Malignant epithelial cells also contributed to ligand expression, albeit to a lesser extent (**Figures 2A, B**). Stromal cells enhance immunosuppression through multiple mechanisms, including physical barrier formation, immune cell reprogramming, and the secretion of immunosuppressive cytokines (27). These functions highlight stromal components as potential therapeutic targets for future research.

Importantly, the expression pattern of TIGIT ligands suggests that the immunosuppressive TME is actively maintained by non-immune stromal components, not just by cancer cells (16, 18). Stromal cells such as fibroblasts and endothelial cells are often spatially proximal to infiltrating T cells and may thus serve as persistent, non-mutated sources of inhibitory ligand signals, contributing to chronic TIGIT engagement and sustained T cell exhaustion (28, 29).

Supporting this, CCI analysis revealed a recurring inhibitory loop in which TIGIT+ CD8+ Tex interact with fibroblasts, endothelial cells, and malignant epithelial cells via NECTIN-2/PVR signaling (**Figure 2C**). These findings highlight a multi-cellular suppressive network that is not confined to tumor–immune cell crosstalk but involves broader tissue architecture within the TME. This CCI cycle likely facilitates the maintenance of T cell dysfunction and represents a barrier to effective anti-tumor immunity.

### 3.3 High TIGIT expression is associated with poor prognosis in CRC

Analysis of RNA-sequencing data revealed that TIGIT mRNA expression was significantly elevated in tumor tissues compared to matched adjacent normal tissues in various of tumors (**Supplementary Figure S2A**) (30). In addition, genomic profiling demonstrated that TIGIT alterations, predominantly in the form of mRNA upregulation and copy number gains, were common among CRC patients, and these alterations were associated with higher TIGIT expression levels (**Figure 2D**) (30). Although TIGIT expression was not significantly different between normal and tumor sites in microsatellite instability (MSI)-low CRC patients, a relatively lower expression was still observed in normal tissues (**Supplementary Figure S2B**).

Survival analysis indicated that high TIGIT expression was significantly correlated with reduced overall survival (OS). CRC patients with high TIGIT expression exhibited markedly poorer OS compared to those with low TIGIT expression (**Figure 2E**).

Interestingly, TIGIT expression appeared to be independent of tumor stage, as no significant differences were observed between early- and late-stage tumors (**Figure 2F**). This suggests that TIGIT upregulation is an intrinsic immunoregulatory feature of the tumor microenvironment, rather than a reflection of tumor burden or progression stage. While TIGIT expression does not vary significantly across tumor stages, its consistent upregulation in the tumor microenvironment suggests its utility as a predictive rather than prognostic biomarker (29). TIGIT^+^ exhausted T cells may indicate a dysfunctional immune contexture that correlates with response to immunotherapy. Further studies are warranted to validate TIGIT as part of a combinatorial marker panel to guide personalized treatment decisions (23).

Receiver operating characteristic (ROC) curve analysis, performed using the R statistical package (31), yielded an area under the curve (AUC) value of 0.61, indicating a moderate predictive potential of TIGIT expression for clinical prognosis in CRC (**Supplementary Figure S2C**) (32). Generally, AUC of 0.5 indicates no discriminatory power, whereas an AUC of 1.0 reflects perfect prediction; thus, an AUC of 0.61 denotes modest discriminatory ability (32). We examined the expression of the TIGIT ligands, PVR and NECTIN-2 in CRC patients. The results demonstrated that there were no significant differences in the expression levels of PVR and NECTIN-2 among CRC patients (**Supplementary Figure S2D**). These findings suggest that while TIGIT expression shows modest prognostic value in CRC, its ligands PVR and NECTIN-2 are not differentially expressed among CRC patients.

Collectively, these findings support the notion that TIGIT expression serves as a negative prognostic biomarker in CRC. Elevated TIGIT expression may contribute to an immunosuppressive tumor microenvironment by interacting with its ligands (e.g., PVR, NECTIN-2) expressed by stromal and malignant cells, thereby promoting T cell exhaustion and enabling tumor immune evasion.

### 3.4 TIGIT^+^ CD8^+^ Tex cells engage inhibitory signaling through NECTIN-2 and PVR

To elucidate the broader CCI network of TIGIT^+^ CD8^+^ Tex cells, we performed CCI analysis using the Smart-seq2 dataset (GSE146771) (20). This analysis identified TIGIT–NECTIN-2 and TIGIT–PVR as dominant inhibitory ligand–receptor axes connecting CD8^+^ Tex cells with both stromal and malignant compartments within the CRC TME (23, 25). The TME comprises a heterogeneous mix of immune cell types, including NK cells, Tregs, CD4^+^ T cells, and CD8^+^ Tex cells (**Supplementary Figures S1A–D**).

**Figure 3:**
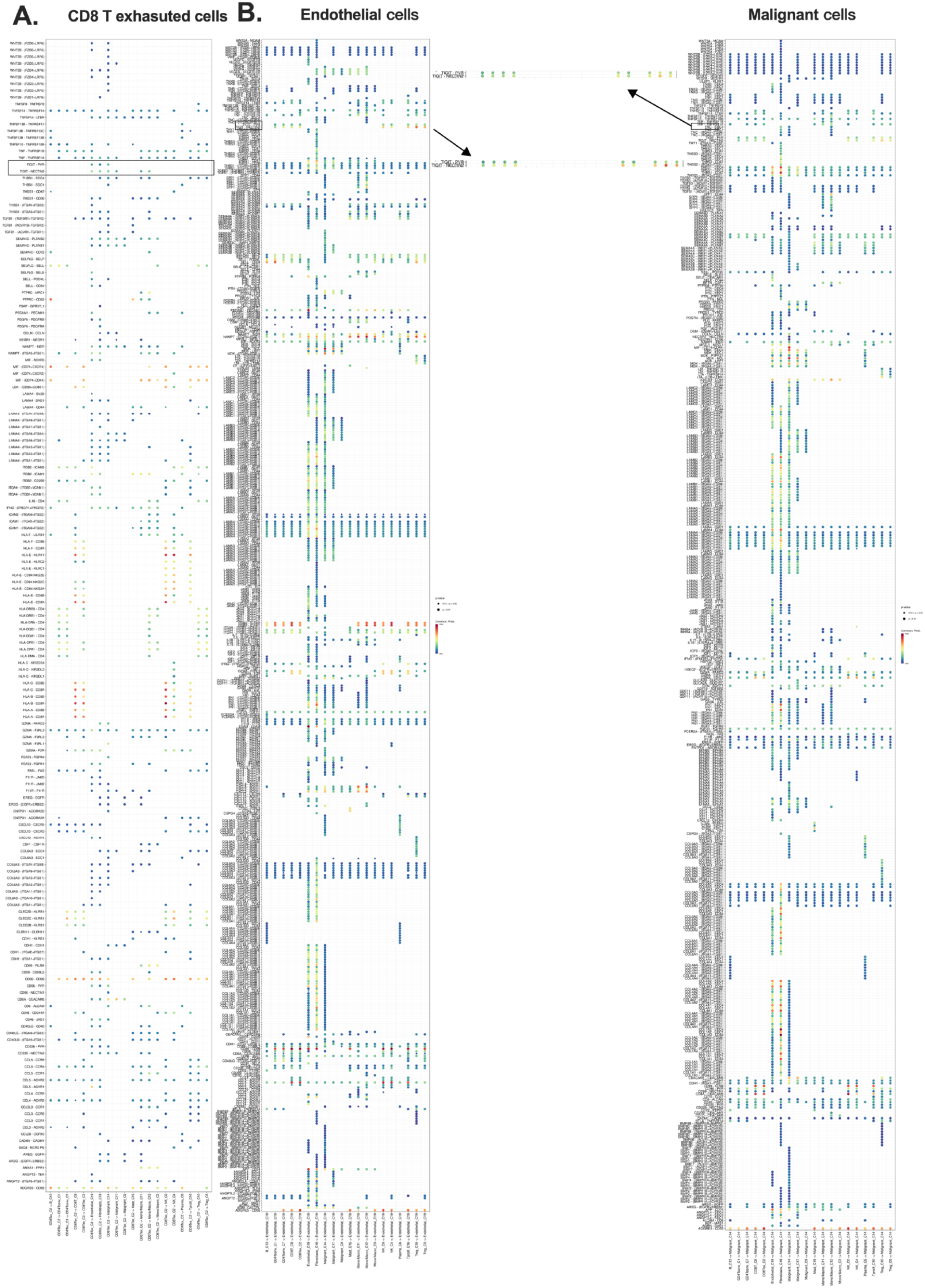
Exhausted CD8^+^ T cells engage in inhibitory crosstalk with stromal and malignant cells. **(A)** CCI source analysis indicating exhausted CD8^+^ T cells as major initiators; **(B)** Target cluster of CD8 Tex include ECs and malignant epithelial cells. Overall survival (OS); cell-cell interaction (CCI); endothelial cells (ECs).

Bubble plots and pie charts revealed that fibroblasts, endothelial cells, and malignant epithelial cells were the primary stromal interactors of CD8^+^ Tex cells, with strong engagement via TIGIT–NECTIN-2/PVR signaling (**Supplementary Figures S1A–D**; **Figures 3A, B**). These findings underscore the coordinated inhibitory interactions between exhausted T cells and non-immune cellular components of the TME, reinforcing the role of these ligand–receptor pathways in facilitating immune suppression and T cell exhaustion (29).

Notably, fibroblasts and endothelial cells emerged as key mediators of T cell dysfunction, predominantly signaling through TIGIT–NECTIN-2/PVR axes (**Figures 3A, B**). These interactions highlight the contribution of both stromal and malignant cells to immune evasion mechanisms, supporting the hypothesis that TIGIT engagement plays a central role in shaping the exhausted T cell phenotype in the CRC microenvironment (**Figures 3A, B**; **Supplementary Figures S5A, B**).

### 3.5 Deconvolution analysis confirms TIGIT–CD8^+^ T cell correlation

To validate the immune cell populations associated with TIGIT expression in CRC, we performed immune deconvolution using two complementary algorithms—CIBERSORT-ABS and QUANTISEQ—on publicly available bulk RNA-seq datasets Timer 2.0 (33). While CIBERSORT-ABS estimates absolute immune cell fractions, QUANTISEQ is specifically optimized for solid tumors with heterogeneous cell populations, thereby providing robust and complementary insights into the tumor immune microenvironment (33, 34).

**Figure 4:**
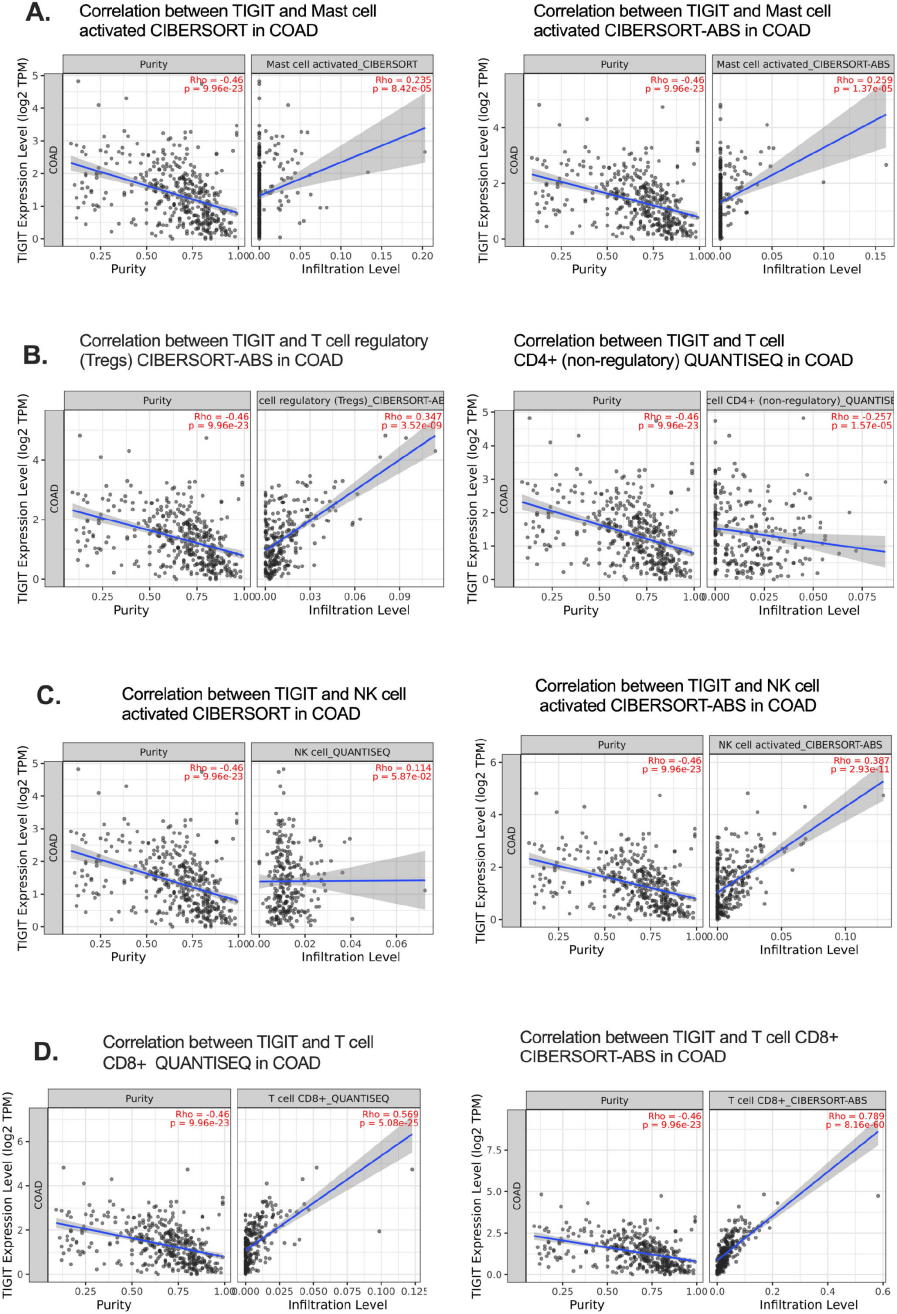
Deconvolution analysis confirms TIGIT–CD8^+^ T cell correlation in CRC. **(A–C)** Correlations of TIGIT expression with immune cell subsets using CIBERSORT-ABS, focusing on activated mast cells, Tregs, and NK cells in CRC; **(D)** Strong positive correlation between TIGIT expression and activated CD8^+^ T cell infiltration.Cell-cell Interation (CCI); ECs (endothelial cells); exhausted T (Tex).

Across both analytical approaches, TIGIT expression showed the strongest positive correlation with CD8^+^ T cell abundance, exceeding correlations observed with other immune cell subsets in the dominant immune population (**Figures 4A–D**). This association supports the hypothesis that TIGIT^+^ CD8^+^ T cells represent a prominent immune phenotype in CRC and reinforces their potential role in mediating T cell exhaustion within the tumor microenvironment. The correlation coefficient (Rho) between TIGIT and CD8^+^ T cells was significantly higher than that of other immune subsets, indicating a preferential enrichment pattern (35).

It is important to acknowledge that the accuracy and resolution of deconvolution algorithms are contingent upon the quality of reference gene signatures, which may vary between datasets and may not fully distinguish exhausted T cell phenotypes. Nevertheless, the consistent results observed across two independent computational frameworks enhance the reliability of our findings and further substantiate the central involvement of TIGIT^+^ CD8^+^ T cells in CRC pathophysiology.

### 3.6 TIGIT^+^ CD8^+^ Tex cells exhibit co-expression with PDCD1 and CXCL13 in CRC patients

**Figure 5:**
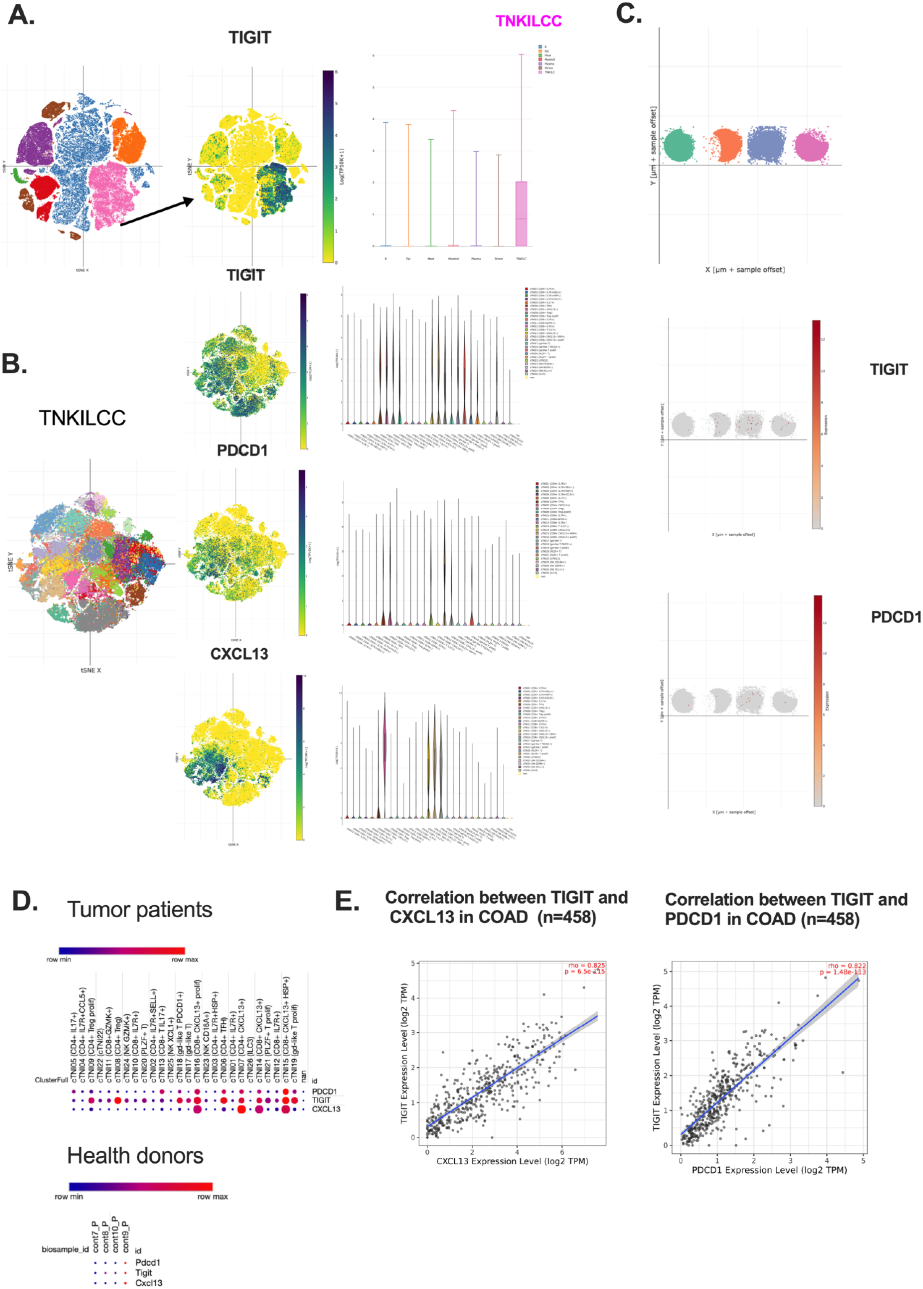
TIGIT^+^ CD8^+^ T cells co-express PDCD1 and CXCL13 and are enriched in CRC patients. **(A)** TIGIT expression distribution across main immune clusters in TIME. **(B)** Spatial localization of TIGIT, PDCD1, and CXCL13 in CD8^+^ Tex cells; **(C)** Comparative expression patterns in healthy donors of TIGIT and PDCD1; **(D)** Co-expression bubble plots for TIGIT, PDCD1, and CXCL13 in tumors and healthy controls; **(E)** Correlation analysis between TIGIT, PDCD1, and CXCL13 in CRC patients (n=458).

To further dissect the immunoregulatory role of TIGIT in the tumor microenvironment, we analyzed scRNA-seq data from CRC patients (GSE178341), analysis revealed that TIGIT expression was predominantly localized to TNKILCC cells, a T cell population enriched in exhausted and cytotoxic features (**Figure 5A**) (36).

Since the expression of TIGIT and PDCD1 on the CD8 T cells are widely used to identify dysfunction or early activated effector T cells in several cancer types such as bladder cancer, these markers alone are insufficient to define terminally exhausted CD8^+^ T cell in CRC (37). Their expression encompasses a heterogeneous population that includes partially exhausted, activated and potentially bystander T cells, limiting their utility as precise prognostic or immunosuppressive markers in CRC TIME (38). CXCL13 has emerged as the critical marker that more specifically delineates the effector-exhausted subset within the PD-1^+^TIGIT^+^CD8 T cell population (39). These CXCL13^+^CD8^+^PD-1^+^TIGIT^+^ T cells, referred to as effector-like CD8⁺ Tex cells, are characterized by robust cytotoxicity, secretion of inflammatory cytokines and chemokines, and strong association with antitumor immune responses, suggesting their involvement in effective tumor control cells (40, 41). In contrast, the CXCL13^-^CD8^+^PD-1^+^TIGIT^+^ T cells exhibit features indicative of terminal exhaustion phenotypes, including diminished functional capacity and reduced responsiveness to tumor antigens, aligning more closely with terminally exhausted CD8^+^ Tex cells.

Therefore, accessing CXCL13 expression within PD-1^+^TIGIT^+^CD8 ^+^ T cells provide a more precise stratification of T cell exhaustion states in the CRC TIME and may serve as a valuable prognostic biomarker. In the ScRNA-seq dataset (GSE1665555), CXCL13 expression was highly enriched in subclusters co-expression TIGIT and PDCD1, particularly in patients with MSI CRC (**Supplementary Figures S4A, B**) (42). Compared to microsatellite stable (MSS) CRC patients, MSI patients(Patient 25) exhibited higher co-expression levels of CXCL13 with TIGIT and PDCD1 in CD8^+^ T cells (14, 43)(**Supplementary Figures S4A, B**). This co-expression pattern suggests a coordinated transcriptional program driving T cell exhaustion in the CRC microenvironment indicate a potential responsiveness to ICB and predict favorable antitumor outcomes.

Upon reclustering this population using refined marker sets, we identified distinct CD8^+^ T cell subsets. Notably, TIGIT, PDCD1, and CXCL13 were co-expressed in the same subclusters, corresponding to CD8^+^ Tex (**Figures 5A, B**). This co-localization suggests a coordinated transcriptional program that defines a dysfunctional T cell phenotype in the CRC microenvironment (44).

Moreover, in the healthy donor samples, it showed markedly lower expression of TIGIT, PDCD1, and CXCL13, indicating that this specific co-expression pattern is tumor-specific (**Figure 5C**). These findings were further supported by t-SNE projections, which revealed overlapping expression domains of TIGIT, PDCD1, and CXCL13 in CRC patients but not in healthy controls (**Supplementary Figures S3A, B**). Boxplot analysis of expression across different cell clusters confirmed that these three genes share similar expression patterns within CRC-infiltrating CD8^+^ T cells (**Supplementary Figures S3C–E**).

This coordinated signaling network, enriched in exhausted CD8^+^ T cells and sustained by signals from both tumor and stromal compartments, underscores a multicellular inhibitory circuit that perpetuates T cell dysfunction (36). These observations support the notion that TIGIT^+^ CD8^+^ T cells function within a suppressive communication loop, positioning TIGIT as a central node in CRC immune evasion (45).

Furthermore, correlation analysis across CRC patient samples demonstrated that TIGIT expression positively correlates with both CXCL13 and PDCD1, reinforcing the existence of a shared regulatory axis in T cell exhaustion (**Figures 5D, E**). In contrast, healthy donors exhibited minimal expression of these genes, further emphasizing their pathological relevance in the tumor context.

### 3.7 Co-culture with colon cancer cells induces CD8^+^ T cell exhaustion

**Figure 6:**
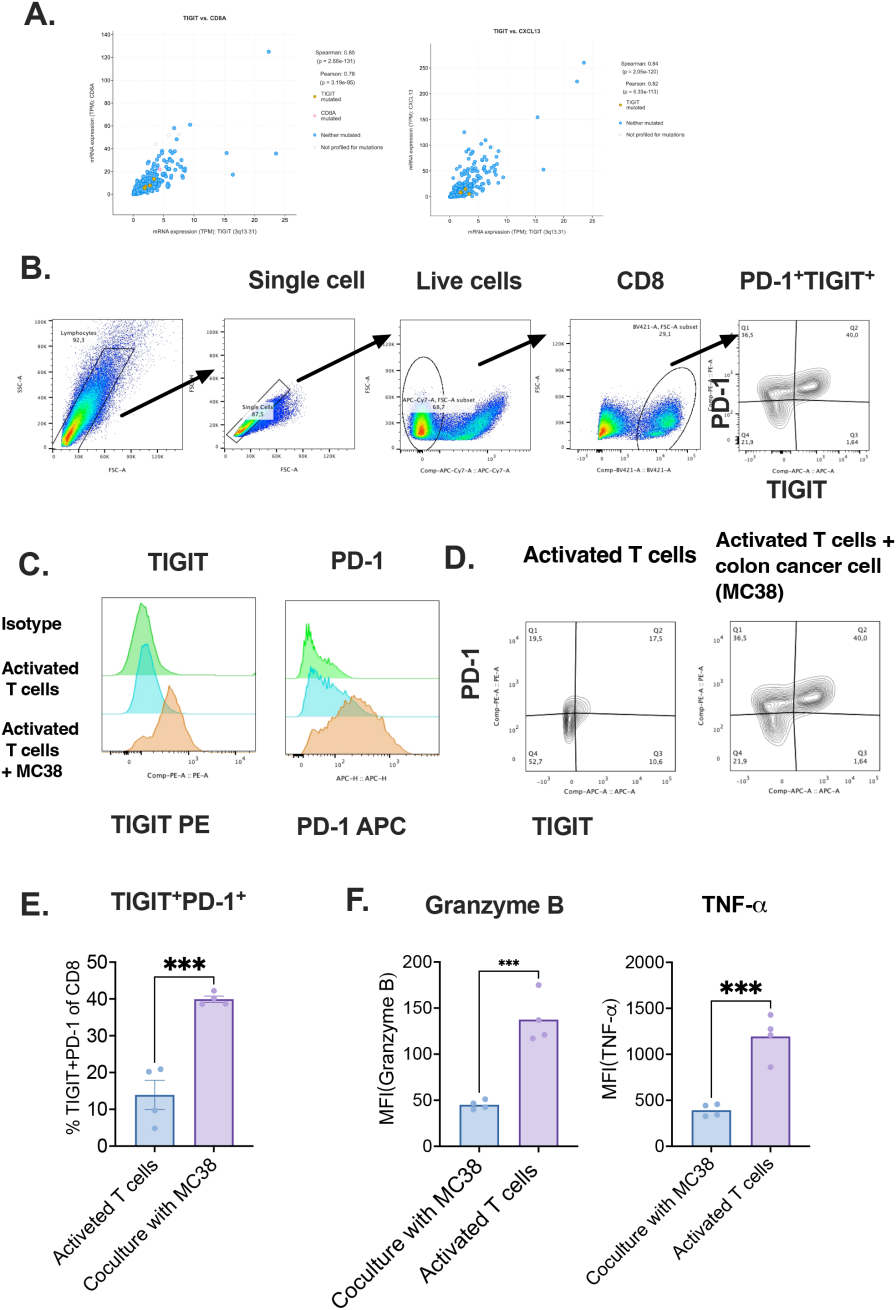
Co-culture with tumor cells induces exhaustion phenotype in CD8+T cells. **(A)** Pearson and Spearman correlation analyses between TIGIT, CD8A, and CXCL13 expression; **(B)** Gating strategy for detecting TIGIT expression in T cells; **(C)** Comparison of TIGIT expression between activated and colon cancer co-cultured CD8^+^ T cells with isotype controls; **(D)** Co-expression of TIGIT and PD-1 in control and tumor-exposed T cells; **(E)** Ratio of TIGIT^+^PD-1^+^ cells among CD8^+^ T cells after *in vitro* activation, with or without tumor cell co-culture; **(F)** Quantification of mean MFI of granzyme B and TNF-α.Tumor immune microenvironment (TIME), Exhausted T(Tex) .

To experimentally validate the transcriptomic observations, murine CD8^+^ T cells were isolated from the spleens of naive mice, activated *in vitro*, and subsequently co-cultured with MC38 colon adenocarcinoma cells (46). Correlation analyses (Pearson and Spearman) demonstrated a strong association between CXCL13 and TIGIT expression in CD8^+^ T cells (**Figure 6A**). Flow cytometric analysis further revealed a significant upregulation of TIGIT and PD-1 in CD8^+^ T cells after co-culture, compared to activated CD8^+^ T cells cultured alone (**Figures 6B–F**). To confirm the exhausted status of CD8^+^ T cells following co-culture with MC38 tumor cells, we also assessed the expression of cytotoxic molecules and cytokines, including granzyme B, and tumor necrosis factor-alpha (TNF-α) (**Figure 6F**). The results showed a significant decrease in granzyme B and TNF-α levels after co-culture, indicating reduced cytotoxic activity.

Notably, the proportion of TIGIT^+^PD-1^+^ double-positive CD8^+^ T cells was significantly elevated under co-culture conditions, indicating the acquisition of an exhaustion-like phenotype upon direct interaction with tumor cells (25). These results strongly support the hypothesis that tumor-derived factors contribute to the induction of immune checkpoint expression, thereby promoting CD8^+^ T cell dysfunction in the colorectal cancer microenvironment.

This *in vitro* evidence aligns with our computational findings and underscores the functional relevance of tumor–T cell interactions in shaping T cell exhaustion. Together, these data highlight a potential mechanistic basis for the observed enrichment of TIGIT^+^PD-1^+^ CD8^+^ T cells in CRC patients and provide a rationale for targeting these pathways therapeutically.

## 4 Discussion

Our study delineates a pivotal role for TIGIT as an immune checkpoint receptor driving CD8^+^ T cell exhaustion within the CRC TIME, with clear translational implications. Through the integration of single-cell and bulk transcriptomic datasets, we demonstrate that TIGIT is highly expressed on CD8 Tex, which co-express PD-1 and the chemokine CXCL13—. This co-expression signature identifies a exhausted yet tumor-reactive T cell subset, indicative of chronic antigen stimulation and reduced effector function. Notably, CXCL13 expression serves as a key marker to further stratify PD-1^+^TIGIT^+^CD8^+^ T cells into distinct exhaustion states. CXCL13^+^ cells are associated with an effector-like Tex phenotype, retaining cytotoxicity and cytokine production, while CXCL13^-^ counterparts exhibit features of terminal exhaustion, including reduced functional capacity and antigen responsiveness. Furthermore, we observed significantly higher CXCL13 expression in CD8^+^ T cells from MSI CRC patients compared to those with MSS tumors. This suggests that CXCL13^+^TIGIT^+^PD-1^+^CD8^+^ T cells may serve as a prognostic biomarker reflecting both immune activation and subtype-specific immune contexture in CRC. These cells are predominantly found in tumor-infiltrating lymphocytes (TILs), offering a more accurate reflection of the TIME (5, 44, 45). While our findings support the prognostic value of TIGIT^+^PD-1^+^CXCL13^+^ CD8^+^ T cells within tumor tissues, future research should also explore non-invasive biomarkers—such as immune signatures detectable in peripheral blood—that could broaden the clinical utility and accessibility of immune monitoring for CRC patients. Furthermore, the following signaling pathways should be explored in future studies to better understand the downstream factors regulating these pathways. This will help clarify the importance of combining targeted therapies with treatments other than immune checkpoint blockade (ICB) (4, 6, 8, 47).Critically, we demonstrate that TIGIT ligands NECTIN−2 and PVR are predominantly presented by stromal fibroblasts, endothelial cells, and malignant epithelial cells, establishing a stable immunosuppressive niche (45). CCI analyses reveal that TIGIT^+^ CD8^+^ Tex cells engage in potent inhibitory crosstalk with both immune (e.g., Tregs) and non−immune compartments via TIGIT–NECTIN−2/PVR axes, highlighting how extrinsic signals perpetuate T cell dysfunction.

Complementary immune deconvolution of bulk RNA−seq data from CRC cohorts confirms a robust correlation between TIGIT expression and CD8^+^ T cell infiltration, further validating TIGIT as a marker of dysfunctional effector populations (45). *In vitro* co−culture assays demonstrate that direct tumor–T cell contact suffices to induce TIGIT and PD−1 upregulation, establishing a causal link between tumor−derived factors and the exhausted phenotype.

Given the link between this cell population and immune suppression or therapy resistance, quantifying TIGIT^+^ Tex cell frequencies—possibly through minimally invasive methods such as peripheral blood sampling or tumor-draining lymph node biopsies—may help predict patient responsiveness to PD−1/TIGIT dual blockade or other immunotherapeutic combinations. A limitation of our current study is the inability to determine whether the observed exhaustion-like phenotype of CD8^+^ T cells in the co-culture system is driven by direct tumor–T cell contact or by tumor-derived soluble factors. Although our findings demonstrate functional impairment of CD8^+^ T cells upon tumor interaction, additional experiments such as transwell assays will be needed to clarify the underlying mechanisms. Future studies addressing this will help delineate the relative roles of contact-dependent versus soluble factor-mediated pathways in driving T cell exhaustion.

Collectively, our findings establish a comprehensive framework for understanding the TIGIT–CD8⁺ Tex axis in CRC and its potential translational relevance. The identification of a TIGIT^+^PD-1^+^CXCL13^+^ CD8^+^ T cell subset—characterized by features of both function and tumor reactivity—highlights a critical population shaped by chronic antigen exposure and enriched particularly in MSI CRC patients. These cells may represent key effectors of antitumor immunity and serve as a biomarker for immune activation and subtype-specific prognosis.

Moving forward, we propose that future studies should validate the prognostic significance of TIGIT^+^PD-1^+^CXCL13^+^ CD8^+^ T cells across independent CRC cohorts, evaluate their detectability in peripheral or cytological specimens, and investigate their dynamic changes in response to TIGIT blockade—alone or in combination with PD-1/PD-L1 inhibitors. These efforts will be crucial for guiding early intervention strategies and advancing precision immunotherapy in CRC.

## Data Availability

The original contributions presented in the study are included in the article/**Supplementary Material**. Further inquiries can be directed to the corresponding authors. The single-cell sequencing data generated in this study have been deposited in the GEO repository (https://www.ncbi.nlm.nih.gov/geo/) under accession numbers GSE146771 and GSE178341.

## Supplementary material

Supplementary Figure 1 Overview of Immune Cell Landscapes in CRC ScRNA-Seq Dataset. **(A, B)** General distribution of major immune cell types; **(C, D)** TIGIT expression mapped across main clusters and subpopulations.Fluorescence intensity (MFI), Tumor necrosis factor (TNF)

Supplementary Figure 2 High TIGIT Expression Associates With Poor Clinical Outcomes. **(A)** TIGIT expression across cancer types with emphasis on CRC; **(B)** TIGIT expression differences between normal tissues and MSI-L CRC tumors; **(C)** ROC curve evaluating TIGIT as a prognostic biomarker in CRC; **(D)** Kaplan–Meier curve showing OS in patients with co-expression of the TIGIT ligands CD155 and CD112

Supplementary Figure 3 Co-expression of CXCL13, TIGIT, and PDCD1 Highlights Exhausted CD8^+^ T Cell States. **(A, B)** t-SNE visualization showing spatial overlap of PDCD1, TIGIT, and CXCL13 in CRC patients; **(C, E)** Boxplots demonstrating expression patterns of TIGIT, PDCD1, and CXCL13 across immune subclusters. Microsatellite instability (MSI), overall survival (OS)

Supplementary Figure 4 Co-localization of TIGIT, PDCD1, and CXCL13 is more pronounced in MSI patients compared to MSS patients, with Patient identified as MSI. **(A)**. scRNA-seq visualization of patient samples, highlighting distinct immune cell clusters; **(B)**. Expression patterns of PDCD1, TIGIT, and CXCL13 across patients, demonstrating stronger co-expression in the MSI patient(patient 25).

Supplementary Figure 5 Ligand–receptor interaction analysis reveals TIGIT–PVR/NECTIN2 signaling between CD8^+^ Tex cells and fibroblasts in CRC samples. (A, B). The source cluster is identified as CD8^+^ Tex cells, and the target cluster as fibroblasts. The analysis highlights ligand–receptor interactions in CRC samples, specifically the engagement of TIGIT on CD8^+^ Tex cells with its ligands PVR and NECTIN- 2.Microsatellite stable (MSS), microsatellite instability (MSI)
