# Supplementary figures and images for: Identification of a TIGIT-expressing CD8+ T cell subset as a potential prognostic biomarker in colorectal cancer
Source: Front Immunol. 2025 Jul 29;16:1626367. doi: 10.3389/fimmu.2025.1626367 (PMC12340724; doi:10.3389/fimmu.2025.1626367)

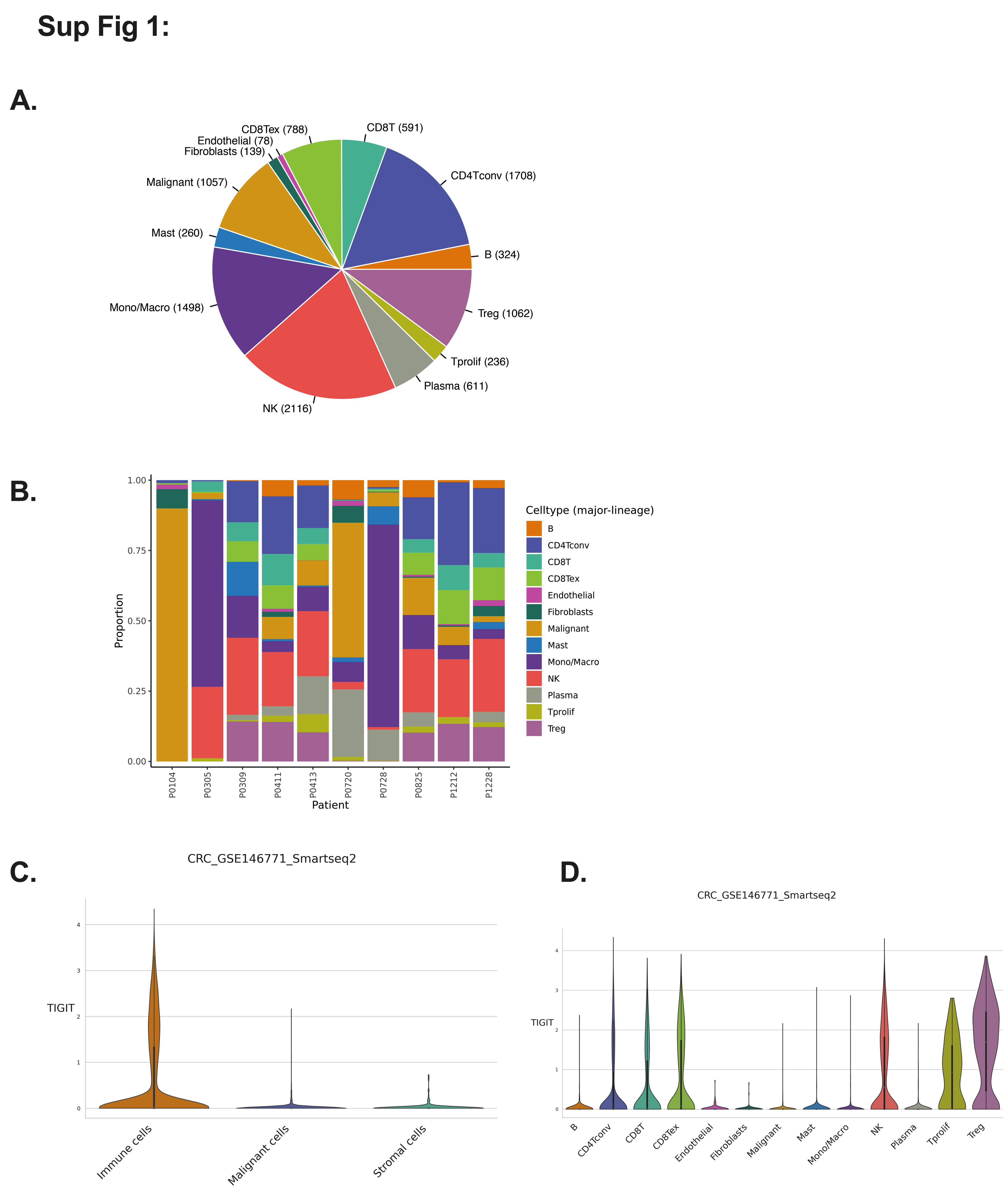

Supplement: Supplementary Figure 1 — Overview of Immune Cell Landscapes in CRC ScRNA-Seq Dataset. (A, B) General distribution of major immune cell types; (C, D) TIGIT expression mapped across main clusters and subpopulations.Fluorescence intensity (MFI), Tumor necrosis factor (TNF) [file Image1.tiff]

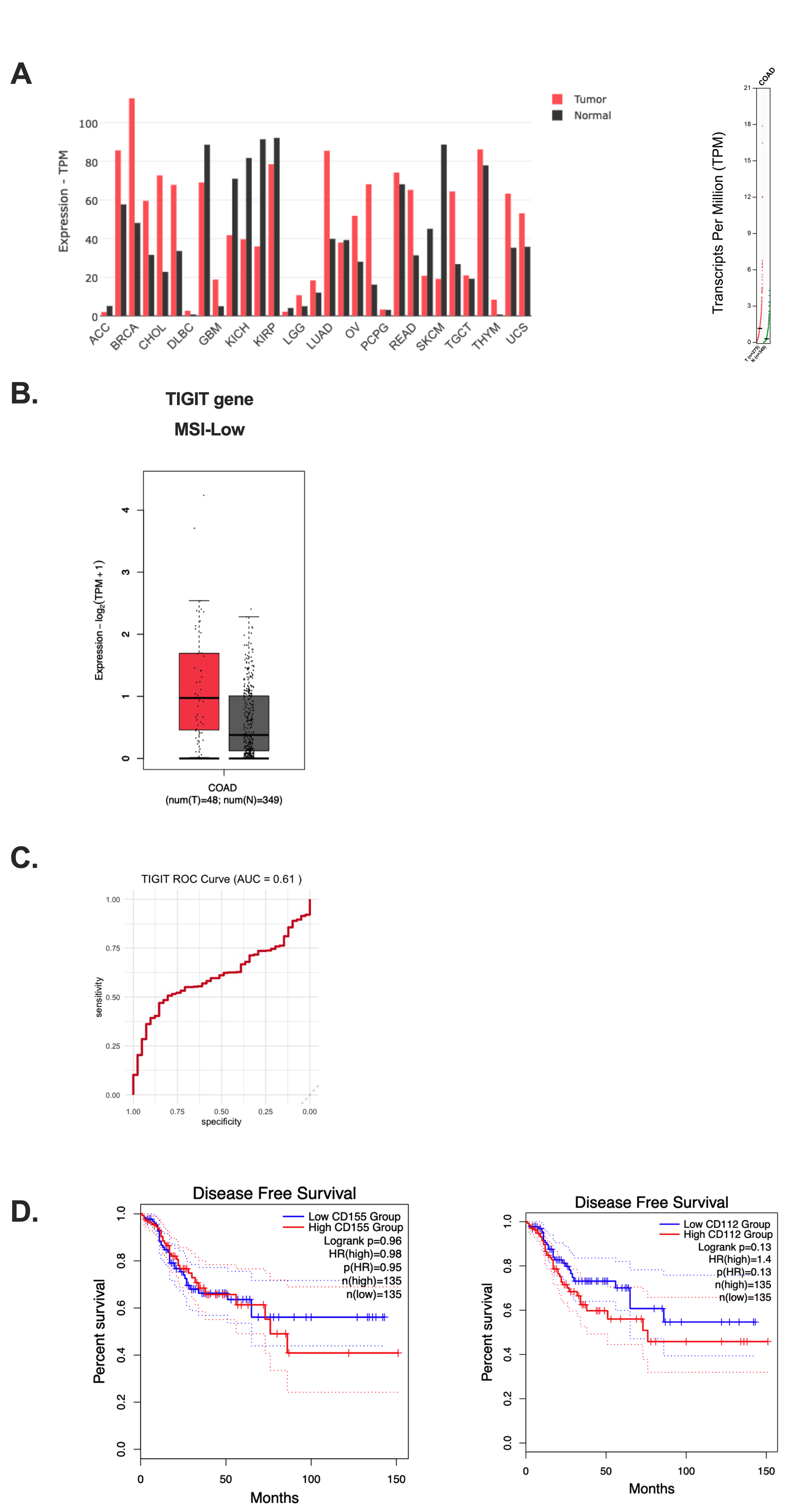

Supplement: Supplementary Figure 2 — High TIGIT Expression Associates With Poor Clinical Outcomes. (A) TIGIT expression across cancer types with emphasis on CRC; (B) TIGIT expression differences between normal tissues and MSI-L CRC tumors; (C) ROC curve evaluating TIGIT as a prognostic biomarker in CRC; (D) Kaplan–Meier curve showing OS in patients with co-expression of the TIGIT ligands CD155 and CD112 [file Image2.tiff]

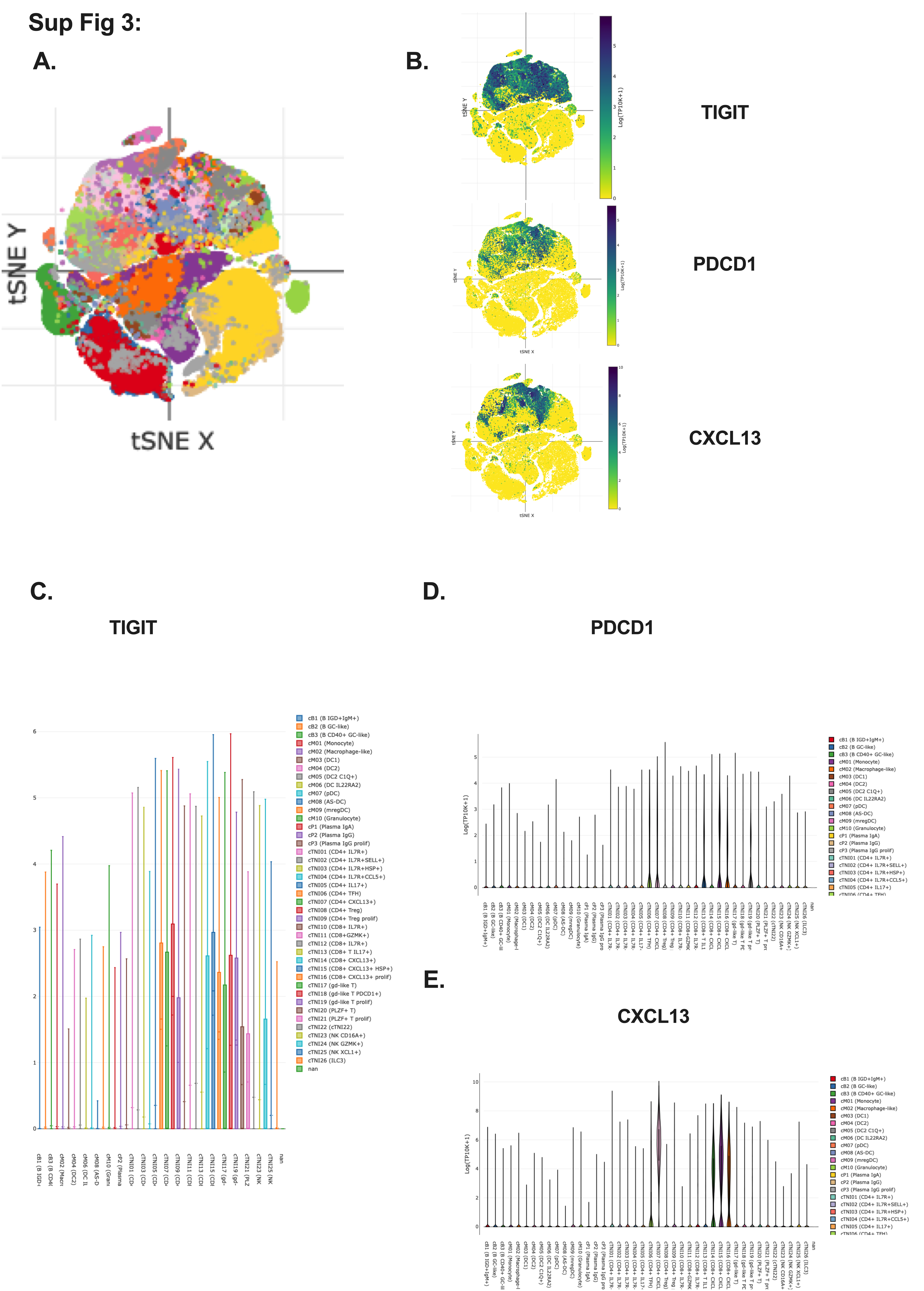

Supplement: Supplementary Figure 3 — Co-expression of CXCL13, TIGIT, and PDCD1 Highlights Exhausted CD8+ T Cell States. (A, B) t-SNE visualization showing spatial overlap of PDCD1, TIGIT, and CXCL13 in CRC patients; (C, E) Boxplots demonstrating expression patterns of TIGIT, PDCD1, and CXCL13 across immune subclusters. Microsatellite instability (MSI), overall survival (OS) [file Image3.tiff]

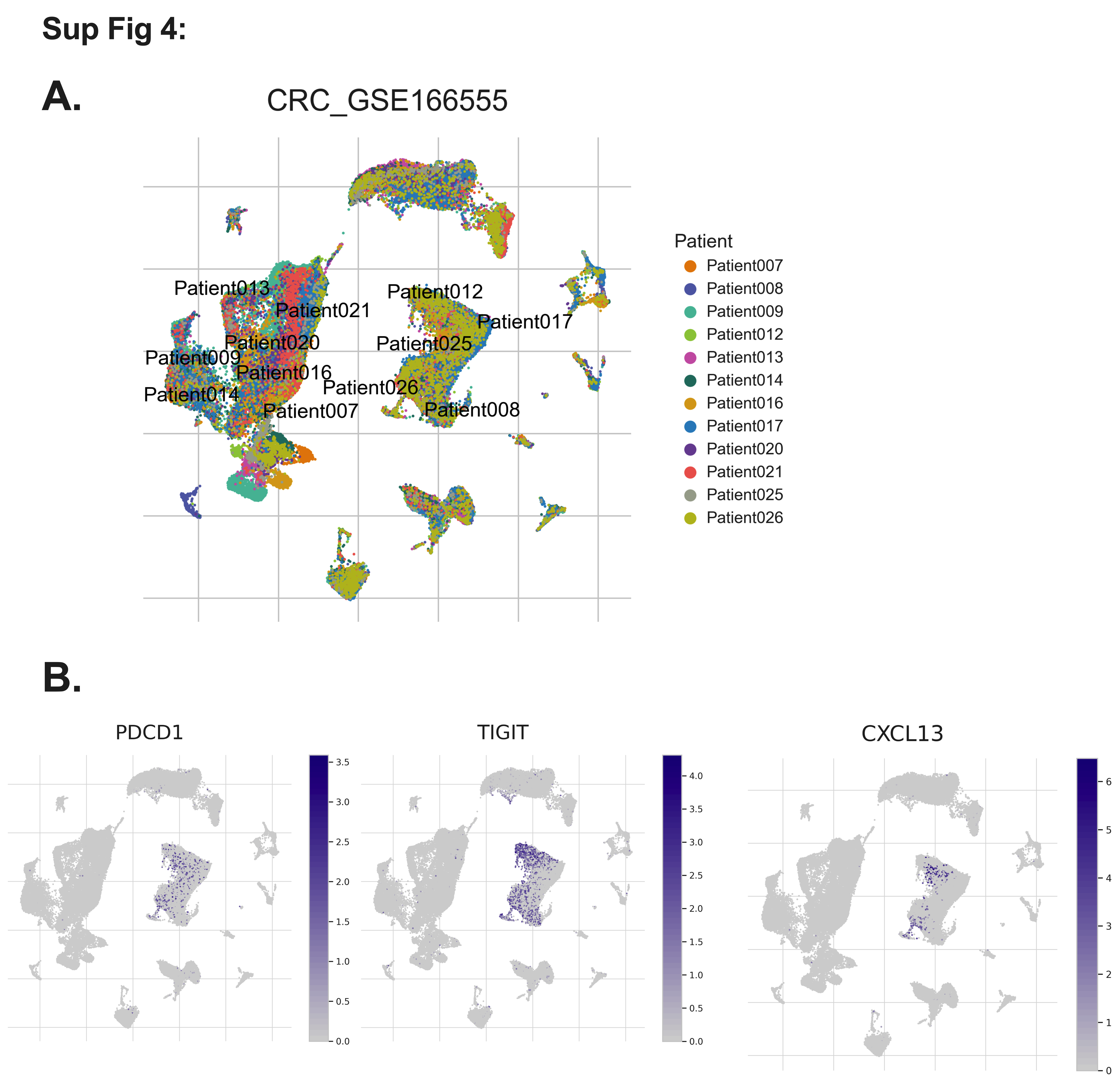

Supplement: Supplementary Figure 4 — Co-localization of TIGIT, PDCD1, and CXCL13 is more pronounced in MSI patients compared to MSS patients, with Patient identified as MSI. (A). scRNA-seq visualization of patient samples, highlighting distinct immune cell clusters; (B). Expression patterns of PDCD1, TIGIT, and CXCL13 across patients, demonstrating stronger co-expression in the MSI patient(patient 25). [file Image4.tiff]

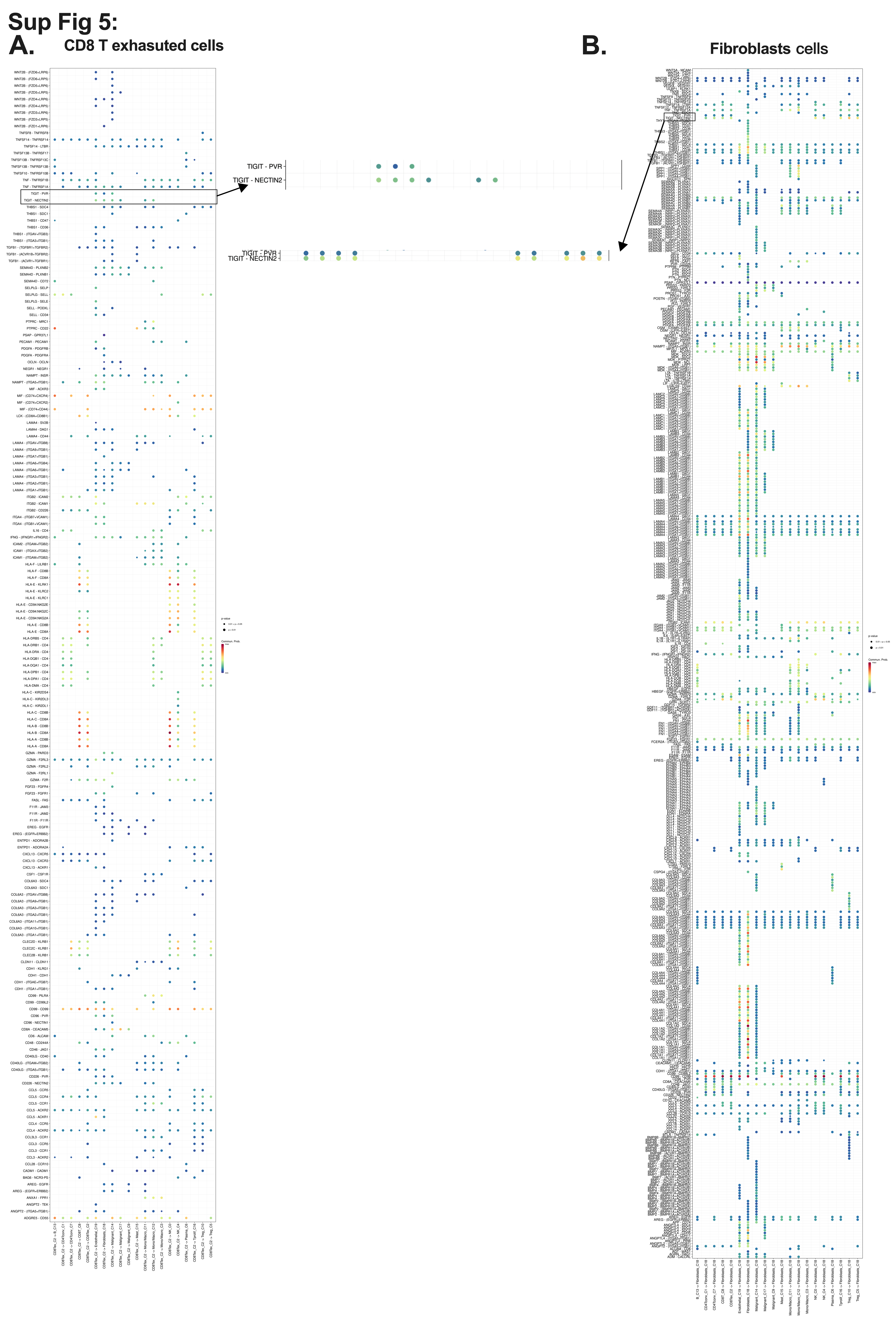

Supplement: Supplementary Figure 5 — Ligand–receptor interaction analysis reveals TIGIT–PVR/NECTIN2 signaling between CD8+ Tex cells and fibroblasts in CRC samples. (A, B). The source cluster is identified as CD8+ Tex cells, and the target cluster as fibroblasts. The analysis highlights ligand–receptor interactions in CRC samples, specifically the engagement of TIGIT on CD8+ Tex cells with its ligands PVR and NECTIN- 2.Microsatellite stable (MSS), microsatellite instability (MSI) [file Image5.tiff]
